# Clinical and prognostic significance of bone marrow abnormalities in the appendicular skeleton detected by low-dose whole-body multidetector computed tomography in patients with multiple myeloma

**DOI:** 10.1038/bcj.2015.57

**Published:** 2015-07-31

**Authors:** Y Nishida, Y Matsue, Y Suehara, K Fukumoto, M Fujisawa, M Takeuchi, E Ouchi, K Matsue

**Affiliations:** 1 Division of Hematology/Oncology, Department of Medicine, Kameda Medical Center, Kamogawa, Japan; 2 Department of Hematology, Respiratory Medicine and Oncology, Division of Internal Medicine, Saga University, Saga, Japan; 3 Department of Cardiology, Kameda Medical Center, Kamogawa, Japan; 4 Department of Radiology, Kameda Medical Center, Kamogawa, Japan

## Abstract

Clinical significance of medullary abnormalities in the appendicular skeleton (AS) detected by low-dose whole-body multidetector computed tomography (MDCT) in patients with multiple myeloma (MM) was investigated. A total of 172 patients with monoclonal gammopathy of undetermined significance (MGUS) (*n*=17), smoldering MM (*n*=47) and symptomatic MM (*n*=108) underwent low-dose MDCT. CT values (CTv) of medullary density of AS⩾0 Hounsfield unit (HU) was considered as abnormal. Percentage of medullary abnormalities and the mean CTv of AS in patients with MGUS, smoldering MM and symptomatic MM were 18, 55 and 62% and −44.5 , −20.3 and 11.2 HU, respectively (*P*<0.001 and *P*<0.001). Disease progression of MM was independently associated with high CTv on multivariate analysis. In symptomatic MM, the presence of abnormal medullary lesions was associated with increased incidence of high-risk cytogenetic abnormalities (34.4% vs 7.7% *P*=0.002) and extramedullary disease (10.4% vs 0% *P*=0.032). It was also an independent poor prognostic predictor (hazard ratio 3.546, *P*=0.04). This study showed that CTv of AS by MDCT is correlated with disease progression of MM, and the presence of abnormal medullary lesions is a predictor for poor survival.

## Introduction

Whole-body low-dose multidetector computed tomography (MDCT) can be used to assess myeloma bone disease, because it can depict lytic bone lesions as well as extramedullary lesions. MDCT also provides information on bone marrow involvement of appendicular skeleton (AS) in patients with monoclonal gammopathy of undetermined significance (MGUS)^1^ or multiple myeloma (MM).^2, 3^ Previous Study with whole body magnetic resonance imaging (MRI) showed that imaging the spine alone in patients with MM would miss 50% of myeloma lesions.^4^ Hillegass *et al.*^5^ reported that extra-axial focal myeloma lesions were missed even in 20% of asymptomatic MM patients if only an axial MRI was performed. Therefore, coverage of extra-spine is necessary for correct staging and for risk stratification in patients with MM. Most of the intramedullary space in the AS is replaced by fatty marrow in healthy adults.^6^ However, it is often infiltrated by neoplastic plasma cells in myeloma patients, and such medullary infiltration proceeded to the destruction of mineralized bone and is usually cleared by successful antimyeloma treatment.^2^ In addition, such medullary abnormalities could be associated with tumor cell burden of myeloma assessed by laboratory parameters,^3^ although little information is available regarding the incidence and prognostic values of such abnormalities in patients with MGUS and MM. We hypothesized that the presence of medullary abnormalities of AS would be associated with the progression of MGUS to MM and of prognostic in symptomatic MM. This study was performed to determine the clinical and prognostic significance of abnormal bone marrow lesions in AS detected by MDCT in patients with MGUS and newly diagnosed MM. To our knowledge, there have been no studies specifically addressing the prognostic significance of medullary abnormalities of AS detected by MDCT in patients with MM.

## Patients and methods

### Patients and initial work-up for myeloma

The study population consisted of 17 patients with MGUS and 155 consecutive patients with newly diagnosed MM (smoldering MM, *n*=47; symptomatic MM, *n*=108) diagnosed between February 2008 and December 2013 at Kameda Medical Center, Kamogawa-shi, Japan. Diagnosis of MGUS, smoldering MM and symptomatic MM was made according to the International Myeloma Working Group criteria and its update.^7, 8^ The patients with symptomatic MM were treated with bortezomib-based combined chemotherapy in the frontline setting. Patients aged ⩽70 years and suitable for high-dose chemotherapy received autologous stem cell transplantation, although treatments varied in dose and interval according to patient's age, vulnerability and the attending physician's preference.^9^ Patients were informed about the benefits and potential risks of MDCT and underwent it parallel to the myeloma work-up. Myeloma work-up included serum and urine protein electrophoreses and immunofixation electrophoresis, quantification of serum immunoglobulin (Ig) and free light chain (κ, λ, and κ/λ ratio), 24-h urinary protein excretion, serum β_2_-microglobulin and lactate dehydrogenase levels. Bone marrow aspiration and biopsy were performed in all patients. Conventional cytogenetic and fluorescence *in situ* hybridization analyses were performed according to the standard method. Chromosomal aberrations, such as 13q and 17p deletions detected by standard karyotyping, or t(4;14), t(14;16) and deletion of 17p detected by fluorescence *in situ* hybridization, were considered as high-risk cytogenetic abnormalities (CA),^10^ while a lack of these abnormalities was considered to indicate standard risk. Written informed consent was obtained from the patients for the study. This study was approved by the institutional review board of Kameda Medical Center in accordance with the Declaration of Helsinki of 1975, as revised in 2008.

### Imaging acquisition and analysis

CT examinations were performed in a non-enhanced manner (no oral or intravenous contrast material application) on an MDCT scanner (Aquilion 64; Toshiba, Tochigi, Japan). A 32 × 1.0-mm^2^ collimation protocol with a 0.5-s rotation time (acquisition time ~45 s) and a constant tube voltage of 120 kV was used. The scan length extended from the skull to the knees (dependent on patient's height, mostly down to the distal epiphysis of the femur). Patients were placed in the supine position, head first, with the arms beside the body for analysis of bone and bone marrow changes. The field of view was adapted to the patient's circumference. Effective radiation doses were generated by an automated tube current modulation protocol (Volume EC; Toshiba) in which the tube current ranged from a minimum of 10 mA to a maximum of 500 mA per rotation with adaptation of tube current in *xy*- and *z*-directions to attenuation of the transradiated object. A convolution kernel (FC03) was used for image reconstruction. To facilitate image analysis, multiplanar reformations in the coronal plane and sagittal plane (section thickness, 3 mm) were calculated using the standard software provided with the scanner. To estimate the effective radiation doses by the MDCT protocol in each patient, dose length products (DLP) and scanning times were provided by the scanner. To determine the effective dose (*E*), we used the following equation: *E*=DLP·E_DLP,_ where E_DLP_ was a region-specific factor normalized to *E*. As MDCT included from the skull to the knees, the mean value (*k*=0.012 mSv/mGy/cm) for all of the included regions was used to estimate patient dose. DLPs provided by the CT scanner were available in 74 patients with symptomatic MM. The median effective dose was 6.9 mSv (5.7–8.7 mSv as 25th–75th percentile).

**Figure 1:**
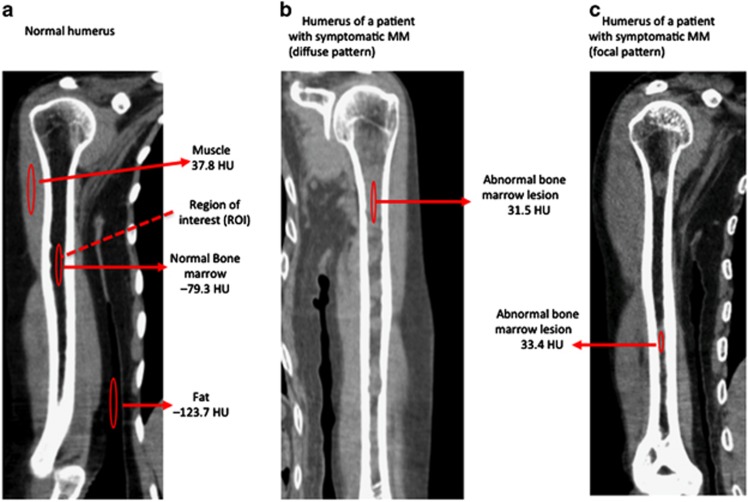
Representative presentation of normal right humeral bone marrow (**a**), abnormal medullary lesions in a patient with MM with diffuse (**b**) and focal pattern (**c**). CTv of the abnormal medullary lesion was measured using a circular region of interest (ROI) and expressed in Hounsfield units.

As the bone marrow of AS is usually replaced by adipose tissue in normal adults and is expected to exhibit lower CT values (CTv) than the density of water (=0 Hounsfield Unit (HU)), bone marrow infiltration with densities in the positive range were considered to indicate myelomatous infiltration. Therefore, medullary lesions in AS with CTv⩾0 HU were considered as abnormal.^3^ Mean CTv of visualized high-density lesions in bony canals of AS were measured using a circular regions of interest. The highest mean CTv of the regions of interest among the bony canals was used as the CTv of the patient. The size of the regions of interest was determined according the size of the respective high-density area. Infiltration of the humeral and femoral bony canals was classified as focal or diffuse according to the pattern of the dense area. The diffuse pattern was defined as homogenous opacity of >90% of the bony canal of any AS, and the focal pattern was defined as the presence of any circumscribed focal high-density area recognized visually. Figure 1 shows representative illustrations of normal right humeral bone marrow and abnormal medullary lesions in a patient with MM with diffuse and focal patterns.

To exclude other causes of bone marrow density change, patients with prior chemotherapy, use of hematopoietic growth factors, active bleeding and disseminated solid cancer were excluded. Patients with systemic amyloid light chain amyloidosis were also excluded from analysis owing to its impact on survival. The CTv of medullary lesions of AS were compared among the patients with MGUS, smoldering MM and symptomatic MM.

### Statistical analysis

Baseline characteristics were analyzed for significance of differences between groups by one-way analysis of variance or Wilcoxon rank-sum test for continuous variables and chi-squared test for categorical variables. Correlations between the CTv and clinical parameters were calculated by Pearson's correlation coefficient. To investigate clinical variables that are potential predictors of the value of CTv in these patients, linear regression models based on the backward Akaike Information Criterion were used. Overall survival (OS) was defined from the date of diagnosis until death from any cause; survivors were censored at the time of last contact. The Kaplan–Meier method was used to estimate OS and comparisons were performed using the log-rank test. Because of the limited number of events, the prognostic implication of abnormal CTv of AS was analyzed in multivariate analysis by adjusting for other known factors, such as age, International Staging System (ISS), high-dose melphalan followed by autologous stem cell transplantation and high-risk CA over the study period.

Data analysis was performed with the Stata SE software ver. 12.1 (Stata Corp., College Station, TX, USA). All statistical test values were two-sided, and *P*<0.05 was taken to indicate significance in all analyses.

## Results

### CTv in patients with MGUS, smoldering MM and symptomatic MM and its correlations with clinical variables

**Figure 2:**
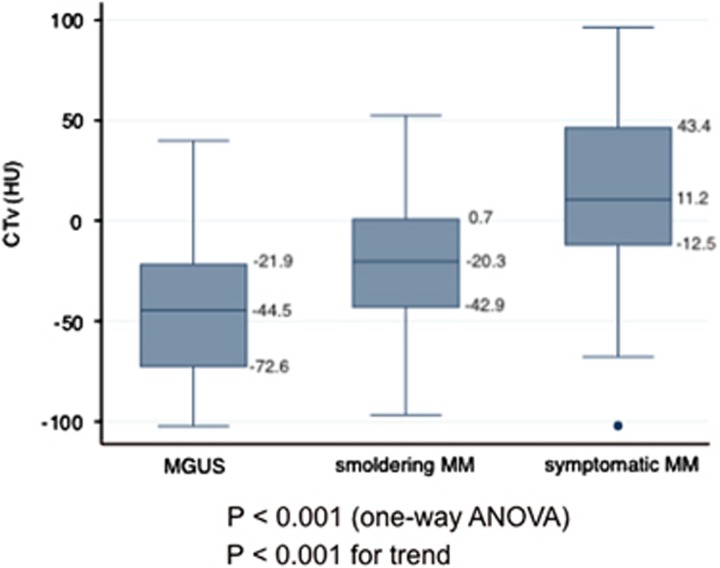
Box plot of CTv in patients with MGUS, smoldering MM and symptomatic MM. ANOVA, analysis of variance.

Table 1 shows the clinical characteristics and abnormal marrow lesions of AS in patients with MGUS, smoldering MM and symptomatic MM in this study. Age and sex were similar between the MGUS, smoldering MM and symptomatic MM groups. The prevalence of the IgG subtype was higher in MGUS compared with MM. The percentages of abnormal medullary lesions in AS in MGUS, smoldering MM and symptomatic MM were 17.6% (3/17), 27.7% (13/47) and 62.0% (67/108), respectively. Of these patients, the respective proportions of diffuse and focal patterns in abnormal lesions were 5.9% and 11.8% in MGUS, 10.6 and 44.7% in smoldering MM and 28.6% and 61.7% in symptomatic MM. Most of the patients with symptomatic MM were Durie–Salmon stage III, and half were ISS III. Figure 2 shows box plots of CTv in patients with MGUS, smoldering MM and symptomatic MM. The mean CTv in patients with MGUS, smoldering MM and symptomatic MM were –44.5 HU (–72.6 to –21.9), –20.3 HU (–42.9 to 0.7) and 11.5 HU (–12.5 to 43.4), respectively (*P*<0.001 by analysis of variance and *P*<0.001 for trend). To estimate independent relevance between CTv and clinical variables, backward Akaike Information Criterion was performed, and the results are shown in Table 2. The results showed that MGUS and smoldering MM were both independently associated with lower CTv compared with symptomatic MM.

### Association of clinical variables in symptomatic MM patients with or without medullary abnormality of AS

Among the 108 symptomatic MM patients, 67 (62%) had abnormal medullary lesions of AS (⩾0 HU). The clinical background of abnormal medullary lesions of AS was evaluated by comparing the two groups with regard to sex, M-protein type, Durie–Salmon stage, ISS, presence of high-risk CA, presence of renal impairment, skeletal-related events and clinical responses (Table 3). There were no differences in sex, M-protein subtype, ISS, renal impairment, skeletal-related events or clinical responses. The proportion of patients with Durie–Salmon stage III was significantly higher in patients with abnormal medullary lesions (82% vs 56% *P*=0.003). The incidence of high-risk CA was significantly increased in patients with abnormal medullary lesions of AS compared with those without medullary lesions (34.4% vs 7.7%, respectively; *P*=0.002), and patients with high-risk CA had significantly higher CTv than those without high-risk CA (mean CTv: 32.9 vs 4.85 HU; *P*=0.003). The study population included 25 patients with high-risk CA, including 8 with 17p deletion, 13 with t(4;14), two with t(14;16) and two with 13q deletion by conventional karyotyping. Of note, extramedullary diseases (EMD) were seen only in patients with abnormal medullary lesions (7/67 vs 0/41; *P*=0.032), and the CTv of patients with EMD was higher than in those without EMD (mean CTv, 46.4 vs 10.7 HU; *P*=0.008).

### Prognostic impact of abnormal medullary lesions of AS in symptomatic MM

**Figure 3:**
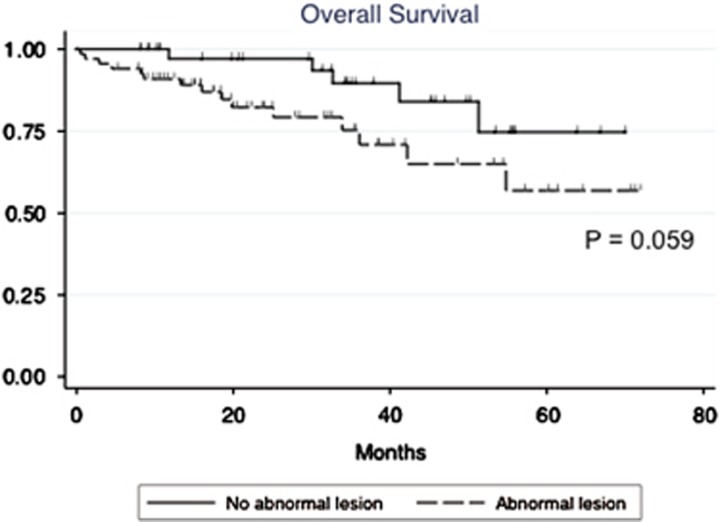
Overall survival of symptomatic MM patients with or without abnormal medullary lesions.

Twenty patients died over a median follow-up of 25.2 months. The Kaplan–Meier curves for OS of both groups are shown in Figure 3. Patients with abnormal medullary lesions showed a lower survival rate than those without such lesions, but the difference did not reach statistical significance (*P*=0.059). However, in multivariate Cox regression analysis, the presence of abnormal medullary lesions was shown to be an independent predictor even after adjustment by age, ISS 3 and high-risk CA, (hazard ratio 3.546; *P*=0.04; Table 4).

## Discussion

Low-dose whole-body MDCT has been explored as an alternative imaging modality to plain radiography for patients with MM.^11, 12^ MDCT has advantages for detecting bone lesions as well as extramedullary lesions.^4, 13^ A recent systematic review of imaging techniques in MM concluded that low-dose MDCT had a similar detection rate to MRI for myeloma bone diseases.^14^ Although detection of bone marrow abnormalities in the axial skeleton is limited by the dense trabeculae in MDCT, it can be easily visualized in bony canals of AS where the trabecular structure is lacking. In addition, its shorter acquisition time (<1 min) represents a major advantage compared with MRI and FDG-PET/CT (positron emission tomography with 2-deoxy-2-[fluorine-18]fluoro-D-glucose integrated with computed tomography), which require more than 1–4 h for whole-body scans. Although a whole-body MRI scanning method with shorter acquisition time (~25 min) could be applied,^15^ lack of suitable MRI scanners and high cost limit its use widespread.

In this study, we focused on the medullary abnormalities of AS in patients with MGUS and MM. As bone marrow of AS in healthy adults is usually replaced by adipose tissue, medullary changes can be visualized more easily than those of central bone such as the pelvic bone and vertebrae, where cancellous structures hamper the recognition of medullary changes by MDCT. Abnormal medullary lesions can be regarded owing to plasma cell infiltration, and this was supported by the positive correlations between CTv and M-protein levels. The percentage of patients with abnormal medullary lesions of AS and CTvs were increased with progression from MGUS to symptomatic MM. Backward Akaike Information Criterion also identified positive correlations between disease status of plasma cell dyscrasia and CTv irrespective of hemoglobin or other laboratory variables. In addition, patients with EMD were included only in the group with abnormal medullary lesions, and those with EMD had higher CTv. These observations indicate that the disease progression is independently associated with an increase in CTv of AS, and CTv in AS reflects the tumor burden of neoplastic plasma cells. Intriguingly, patients with abnormal medullary lesions showed higher incidences of high-risk CA, such as t(4;14), t(14;16) and deletion of 17p detected by fluorescence *in situ* hybridization, compared with those without abnormal medullary lesions. This suggests that medullary abnormalities in AS in myeloma patients imply more aggressive behavior of neoplastic plasma cells. Although FDG-PET/CT can also depict bone marrow of AS and identify hypermetabolic extramedullary lesions as well as medullary lesions in a single procedure, myelomatous lesions with non-proliferative dormant clones are usually hypometabolic and may be overlooked on FDG-PET/CT.

The prognostic impact of medullary abnormality of AS in patients with symptomatic MM has not been described previously. With a median observation period of 25.2 months, the OS of patients with abnormal medullary lesions appeared shorter compared with those without medullary lesions, and the presence of abnormal medullary lesions was shown to be an independent prognostic factor for OS on multivariate analysis. Our observations suggest that the presence of medullary lesions in AS could be regarded as an indicator of high tumor burden, aggressive disease and poor prognosis. Although high-risk CA did not show prognostic value on OS in our analysis, this may be explained by the use of bortezomib dexamethasone that improved the outcome of patients with t(4;14) accounting for 52% (13/25) of the patients with high-risk CA^16^ in this study.

We could not explore the association between the development of skeletal events and the presence of medullary abnormalities in this study owing to the short follow-up period. In addition, patients with lytic cortical lesions detected by MDCT at diagnosis received local irradiation as well as antimyeloma therapy. These patients rarely developed skeletal events during the observation period. Most lytic cortical lesions in AS were preceded by abnormal medullary lesions, as indicated in Figure 1b, although it remains unclear which patients with abnormal medullary lesions in AS develop lytic cortical lesions.

Although bone marrow abnormalities of AS were associated with laboratory variables and myeloma progression, the present study had several limitations, including its retrospective nature, relatively short follow-up duration and non-uniform treatment. We did not estimate progression-free survival owing to the variety of treatment regimens and doses of antimyeloma therapies used.

In conclusion, our results indicated that the presence of abnormal bone marrow lesions in the AS detected by MDCT was associated with high tumor burden, advanced disease stage and poorer prognosis in patients with symptomatic MM.

## Figures and Tables

**Table 1:** Patient characteristics and abnormal bone marrow lesions in patients with MGUS, smoldering MM and symptomatic MM

		*MGUS (*n=*17)*	*Smoldering MM (*n=*47)*	*Symptomatic MM (*n=*108)*	P-*value*
Median age (range)		72 (53–95)	71 (43–92)	74 (44–89)	0.72
Male sex (%)		10 (58.8)	26 (55.3)	59 (54.6)	0.997
M-protein-type IgG (%)		15 (88.2)	31 (66.0)	59 (54.6)	0.022
Non-IgG (%)		2 (11.8)	16 (34.0)	49 (45.4)	
D–S stage III (%)		−	1 (2.1)	78 (72.2)	<0.001
ISS III (%)		−	9 (19.0)	55 (50.9)	0.001
Abnormal medullary lesions (%)		3 (17.7)	13 (27.7)	67 (62.0)	<0.001
Diffuse (%)		1 (5.9)	5 (10.6)	30 (28.6)	0.087
Focal (%)		2 (11.8)	21 (44.7)	66 (61.7)	0.08

Abbreviations: D–S, Durie–Salmon, IgG, immunoglobulin G; ISS, International Staging System; MGUS, monoclonal gammopathy of undetermined significance; MM, multiple myeloma.

**Table 2:** Correlations of clinical variables correlated and CTv by linear regression model using backward AIC

	*Standardized beta coefficient*	t-*value*	P*-value*
Age	–0.133	–1.857	0.065
Hemoglobin	–0.172	–2.389	0.018
M-protein levels	0.23	2.856	0.005
MGUS	–0.238	–3.502	0.0006
Smoldering MM	–0.203	–2.575	0.011

Abbreviations: AIC, Akaike Information Criterion; CTv, CT value; MGUS, monoclonal gammopathy of undetermined significance; MM, multiple myeloma.

**Table 3:** Characteristics of patients with or without abnormal medullary lesions (CTv⩾0) of AS

	*No medullary lesions*	*Abnormal medullary lesions*	P-*value*
	*(*n=*41)*	*(*n=*67)*	
Median age	74	71.4	0.195
Male sex	21 (51.2)	38 (56.7)	0.578

*M-protein type*			
IgG	19 (46.3)	40 (59.7)	0.176
IgA	15 (36.6)	16 (23.9)	0.157
			
Durie–Salmon III	23 (56.1)	55 (82.1)	0.003
ISS III	18 (43.9)	37 (55.2)	0.253
High-risk CA	3 (7.7)	22 (34.4)	0.002
Renal dysfunction	5 (12.2)	16 (23.9)	0.136
SREs	17 (47.2)	32 (52.5)	0.647
EMD	0 (0)	7 (10.4)	0.032
HDM+ASCT	10 (24.4)	19 (28.4)	0.204

*Clinical response*			
CR	16 (48.5)	22 (38.6)	0.36
⩾VGPR	20 (60.6)	31 (54.4)	0.566

Abbreviations: AS, appendicular skeleton; ASCT, autologous stem cell transplantation; CA, cytogenetic abnormality; CR, complete response; CTv, CT value; EMD, extramedullary disease; IgG, immunoglobulin G; HDM, high-dose melphalan, ISS, International Staging System; SRE, skeletal-relating event; VGPR, very good partial response.

**Table 4:** Univariate and multivariate analysis for overall survival according to the known prognostic factors

	*Univariate*			*Multivariate*		
	*HR*	*95% CI*	P*-value*	*HR*	*95% CI*	P*-value*
Age	1.063	1.007–1.121	0.026	1.096	1.011–1.188	0.026
ISS 3	3.3	1.854–5.876	<0.001	2.486	1.305–4.737	0.006
High-risk CA	2.125	0.868–5.204	0.164	1.135	0.362–3.561	0.827
HDT+ASCT	0.353	0.820–1.527	0.164	1.073	0.175–6.586	0.94
Abnormal CTv	2.575	0.930–7.131	0.069	3.546	1.059–11.87	0.04

Abbreviations: ASCT, autologous stem cell transplantation; CA, cytogenetic abnormality; CI, confidence interval; CTv, CT value; HDT, high-dose therapy; HR, hazard ratio; ISS, International Staging System.
